# One‐stage combined approach en bloc vertebrectomy for primary Ewing's sarcoma of mobile spine in an adult patient: 3 years following aggressive surgery for a rare entity

**DOI:** 10.1002/ccr3.8170

**Published:** 2023-11-15

**Authors:** Mohamed Amine Gharbi, Houssem Eddine Chahed, Sana Ben Slama, Anis Teborbi, Ramzi Bouzidi, Mouadh Nefiss

**Affiliations:** ^1^ Department of Orthopedic and Trauma Surgery Mongi Slim Marsa University Hospital Center Tunis Tunisia; ^2^ Faculty of Medicine of Tunis University of Tunis El Manar Tunis Tunisia; ^3^ Department of Anatomical Pathology Mongi Slim Marsa University Hospital Center Tunis Tunisia

**Keywords:** en bloc vertebrectomy, Ewing's sarcoma, surgery, thoracic spine

## Abstract

**Key Clinical Message:**

Currently, there is no consensus on the optimal management of nonmetastatic Ewing's sarcoma of the mobile spine. However, associated to chemotherapy, aggressive surgery with en bloc wide resection seems to improve local control and survival.

**Abstract:**

Primary Ewing's sarcoma (EWS) of non‐sacral spine is extremely rare, especially in middle‐age. Therapeutic strategy aims: to large tumor resection, to provide spine stability and to avoid recurrence through chemo and radiotherapy. We report a case of thoracic spine EWS in an adult treated by combined approach en bloc vertebrectomy.

## 1 INTRODUCTION

Primary Ewing's Sarcoma (EWS) of the spine is extremely rare. It accounts for only 3.5%–14.9% of all primary bone sarcomas.^1^, ^2^ It is characterized by a high proliferative and invasive potential and a confusing variety of imaging manifestations in adult patients.^3^ Nonspecific signs are often in the first place and the delay from the onset of symptoms to the diagnosis can take months which can worsen the prognosis.^4^ Because of the rarity of the spinal localization of primary EWS in adults there is not a well‐coded management protocol and a multitude of therapeutic strategies that mimic the management of EWS of the appendicular skeleton have been employed. The therapeutic goal is a complete tumor removal and spinal column stability restoration.^2^ The publications of Weinstein, Boriani and Biagini and their surgical staging system (WBB) have been of great interest in this field.^5^, ^6^ We report a rare localization of solitary thoracic spine EWS in an adult patient that was treated with en bloc vertebrectomy.

## 2 CASE REPORT

**FIGURE 1:**
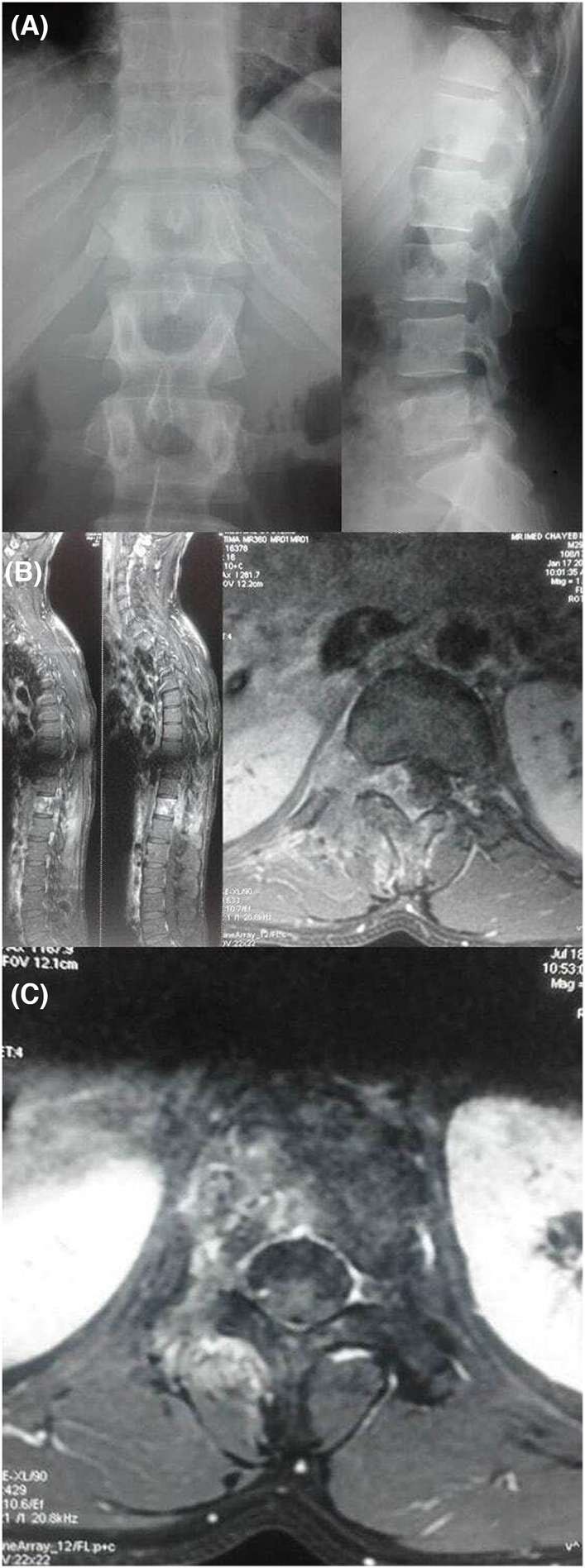
(A) Standard X‐ray (antero‐posterior and lateral view) showing a disappearance of normal contours of the right pedicle of T12. (B) MRI (sagittal and axial view) revealing a vascularized mass affecting the right hemivertebral body and posterior arch of T12 with paravertebral soft tissue extension and spinal cord compression. (C) Axial MRI view showing tumor regression after neoadjuvant chemotherapy.

**FIGURE 2:**
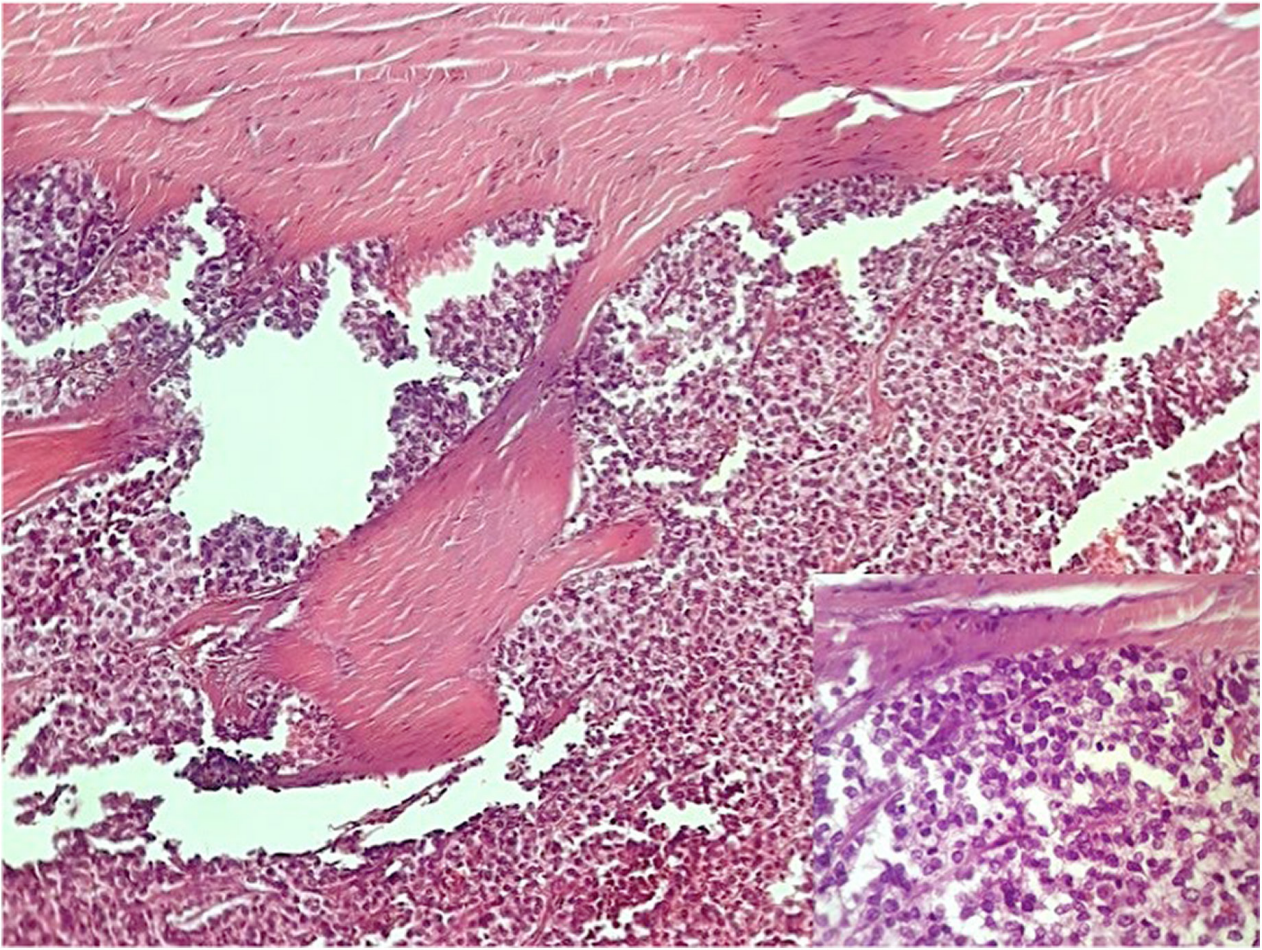
Microscopic examination. Classical Ewing's Sarcoma: Sheets of monotonous small round blue cells with minimal amounts of stroma. Presence of peripheral bone at the top (hematoxylin–eosin: HEx100). Cartridge: Cellular details with round nuclei and minimal cytoplasm (hematoxylin–eosin: HEx200).

A 30‐year‐old man presented with a one‐year history of a progressive, worsening back pain associated to a right intercostal neuralgia, no motor weakness or sphincter disturbance were found. There was no history of fever or trauma. Blood tests were normal and the X‐ray rechecks after making the diagnosis showed an unobvious suspect image at the level of the right pedicle of the 12th thoracic vertebra (T12) with disappearance of its normal contours (Figure 1A). Magnetic resonance imaging revealed a vascularized mass affecting the right hemivertebral body and posterior arch of T12 with paravertebral soft tissue extension. The spinal cord was mildly compressed (Figure 1B). An extension assessment concluded that it is a primary localization. We performed a computerized tomography guided biopsy and the diagnosis of classical EWS was confirmed by histopathology and immunohistochemistry showing monotonous small round blue cells with minimal amounts of stroma, regular round nuclei and minimal cytoplasm, which were strongly positive to CD99 (Figure 2). Cytogenetic analysis technics were not disposable to evaluate transcript fusion type.

After receiving neoadjuvant chemotherapy based on The EUROpean Ewing tumor Working Initiative of National Groups 1999 (EURO‐E.W.I.N.G. 99) protocol including six courses of Vincristine, Ifosfamide, Doxorubicin, and Etoposide (VIDE), a new MRI showed vertebral and paravertebral soft tissue mass regression with longest axial diameters diminution of over 50% (Figure 1C). According to the WBB surgical staging system, sectors included in the axial T12 imaging were 1–6 (part of the body and part of the left posterior arch) and layers of tissue penetration were A to D.

**FIGURE 3:**
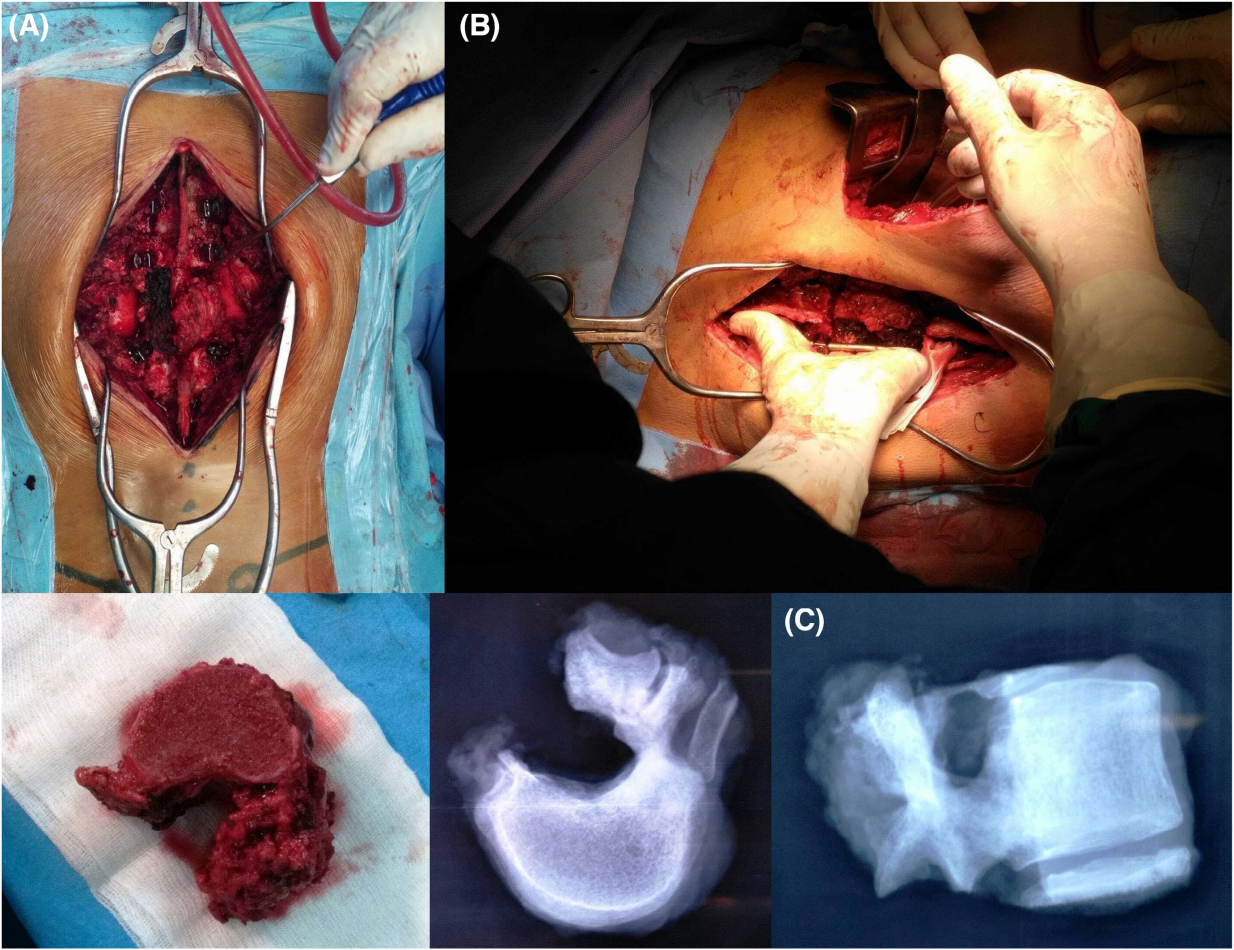
(A) Posterior approach with pedicle screw insertion and release of the spinal cord. (B) En bloc vertebrectomy of T12 through the combined approach. (C) Photo and X‐ray of T12 after extraction.

We performed an en bloc vertebrectomy through a double approach surgery: firstly a posterior approach with pedicle screw insertion (two levels above and two levels below), remove of the healthy part of the posterior arch which allow to release the spinal cord from the tumor pseudocapsule, ligation and section of T12 roots (Figure 3A). Secondly a thoraco‐phreno‐lombotomy to separate the anterior part of the tumor and control the segmental artery. The separation was made by a Gigli saw and the extraction of the vertebra released from all its attachments was possible through a simple rotation of the specimen (Figure 3B,C). Reconstruction was performed with interbody free non‐vascularized fibular bone grafting stabilized by the posterior instrumentation.

Histopathological examination of the resected specimen confirmed classical EWS with clear resection margins and good response to chemotherapy with 97% tumor necrosis rate.

**FIGURE 4:**
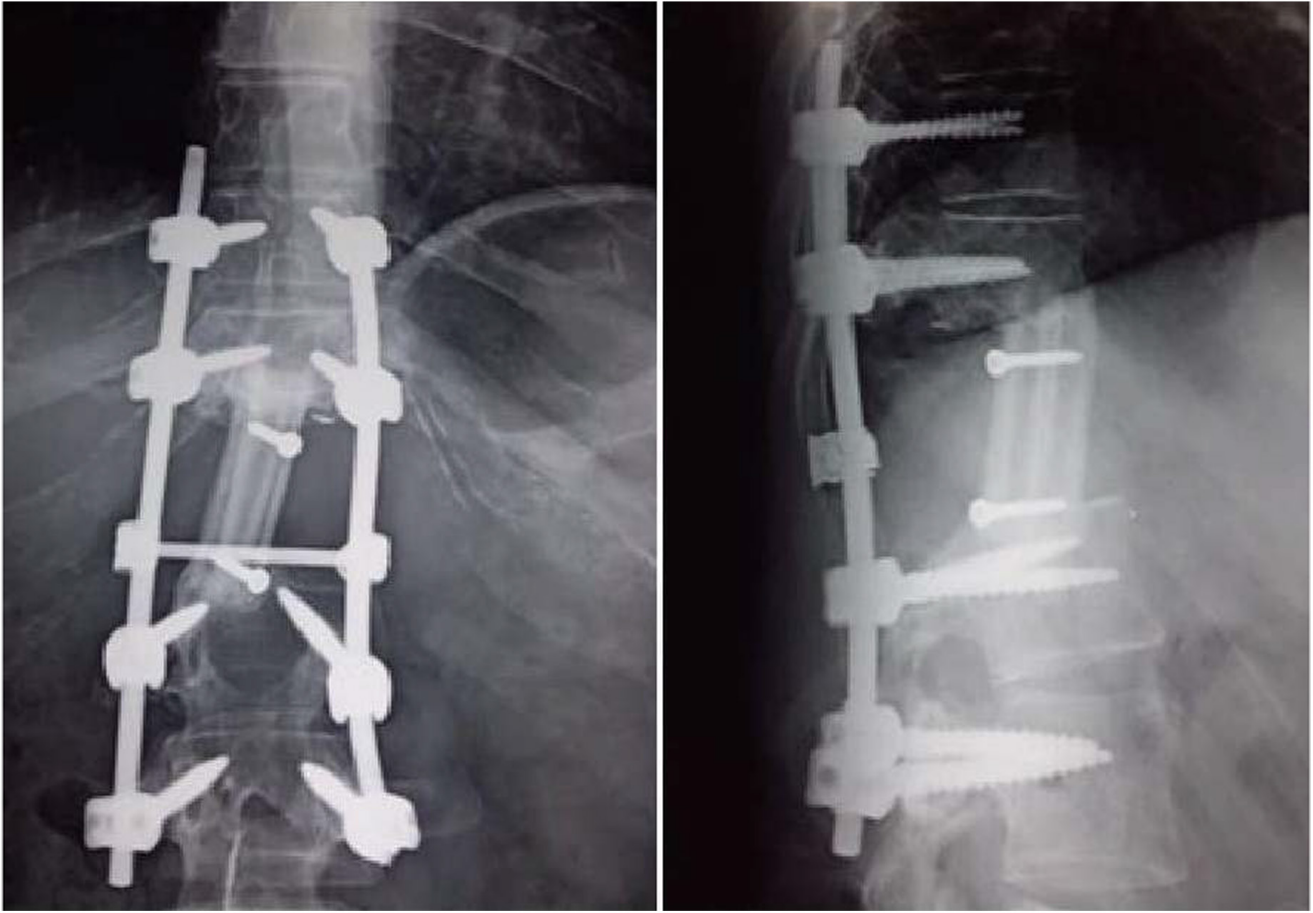
Reconstruction of the anterior column by interbody free non‐vascularized fibular bone grafting stabilized by the posterior instrumentation: X‐ray at 3 years follow‐up showing consolidation without kyphosis.

Postoperative course was uneventful. After 6 weeks of the surgery, the patient started adjuvant chemotherapy and completed treatment with no evidence of disease on reevaluation at the end of therapy. Three years after surgery, the patient is surviving without neurologic deficit, X‐rays revealed consolidation (Figure 4) and with no evidence of recurrence.

## 3 DISCUSSION

EWS remains an enigmatic and particular malignant tumor 100 years after its discovery. Spinal involvement most commonly results from metastasis in advanced stages of the disease, while EWS originating from the spine is rare and extremely rare if the sacrum is excluded.^2^, ^3^ Diagnosis could be quite difficult due to an insidious onset, nonspecific symptoms, and misinterpreted images. Thus, symptoms may not be present until neurological deficits occur and diagnosis suspicion may be after several consultations which increase doubts.^7^, ^8^ The average delay from the onset of symptoms to the diagnosis has been reported to be 34 weeks all locations combined.^9^ In our case the delay was 48 weeks. That's why approximately 25% of patients present with metastatic disease at diagnosis; fortunately this was not the case of our patient.^3^

Definitive diagnosis requires cytological, immunohistochemical (CD99), and cytogenetic analysis of a pathologic specimen. The translocation involving chromosome 22 is identified in more than 90% of cases, and it is the landmark to differentiate EWS from other small, round blue cell tumors.^3^, ^10^

Currently, early diagnosis and multimodal treatment combining surgery, chemotherapy, and local radiation therapy increases the chance of a successful outcome.^11^ Indelicato et al,^12^ in a review have reported a five‐year overall survival rate of 71% and local control rate of 89% for nonmetastatic spinal and paraspinal EWS. However, when compared to other sites of occurrence, prognosis of EWS of the spine remains worse.^13^

Initial chemotherapy and local radiotherapy might be administered before surgery with the aim to shrink bulky and unresectable tumors, to eradicate micrometastases and for acute relief of epidural compression, but it should be noted that there is a variable sensitivity to radiation and chemotherapy due to biological heterogenecity.^1^

Although, local radiotherapy had a considerable place in the management of locally extended or unresectable spine EWS especially with epidural compression or with poor chemotherapy response, many authors emphasized the local control superiority of wide surgical resection R0 in patients with good response following induction chemotherapy. In the other hand, safe margins tumor resection, when technically achievable, allows to avoid epidural and local skin and soft tissues potential complications of radiotherapy.^2^, ^11^, ^12^

In fact, during the last two decades, the outcome in patients with localized disease has improved through an aggressive surgery known as en bloc vertebrectomy by combining anterior and posterior approach or from a single posterior approach as described by Tomita and al.^14^

Boriani et al^6^ described three major methods of performing en bloc excisions in the thoracolumbar spine: vertebrectomy if the tumor is confined to zones 4–8 or 5–9; sagittal resection when the tumor occupies zones 3–5 or 8–10 and resection of the posterior arch when it is located between the zones 10 and 3 according to their surgical staging system.

However, en bloc resection is a highly demanding procedure that must be carefully planned and the greater surgical risk can be accepted only if it offers a safer result and is performed by specialized surgical and anesthesiology teams.^5^

Among the difficulties to be considered is the release of the spinal cord if the tumor is expanding to layer D according to WBB staging system. In this case theoretical safe margin should include the dura in the resection specimen but the cost‐to‐benefit ratio of such procedure should be carefully evaluated. Boriani and his collaborators have shown that a simple release of the dura without resection can be accepted and without proven consequence on the risk of recurrence.^6^, ^13^

Compared to cases where only decompression or lesionectomy was done, patients who underwent en bloc spondylectomy had a lower recurrence rate.^8^, ^15^ This is why, whenever possible, surgical en bloc wide resection with an anterior column reconstruction is preferable in order to obtain a better oncological control and a better preservation of the spine biomechanics.^2^, ^3^, ^16^

## 4 CONCLUSION

Primary EWS of the thoracic spine is an extremely rare tumor. It is a challenging disease not only to treat but also to diagnosis. A high index of suspicion is needed in patients who present with few or nonspecific symptoms. Early diagnosis is essential to obtain better results and improve prognosis. In localized forms of the tumor, multi modal treatment involving neoadjuvant chemotherapy, wide en bloc vertebrectomy with solid reconstruction and adjuvant radiotherapy and chemotherapy is required for patient outcome and satisfactory quality of life.

## AUTHOR CONTRIBUTIONS

**Mohamed Amine Gharbi:** Data curation; formal analysis; investigation; methodology; software; writing – original draft. **Houssem Eddine Chahed:** Resources; software. **Sana Ben Slama:** Resources; software. **Anis Teborbi:** Supervision; validation; visualization. **Ramzi Bouzidi:** Project administration; supervision; validation; visualization. **Mouadh Nefiss:** Conceptualization; investigation; methodology; software; writing – review and editing.

## FUNDING INFORMATION

The author(s) received no financial support for the research, authorship and/or publication of this article.

## CONFLICT OF INTEREST STATEMENT

The authors declare having no conflicts of interest regarding this article.

## CONSENT

Written informed consent was obtained from the patient to publish this report in accordance with the journal's patient consent policy.

## Data Availability

None.

## References

[1] Cherraqi A , Lemrabet A , Dokal ID , et al. Primary Ewing's sarcoma of the spine: about a case. Glob Pediatr Health. 2022;17(9):2333794X221123874. 10.1177/2333794X221123874 PMC9677177 36420454

[2] Zhang J , Huang Y , Lu J , et al. Impact of first‐line treatment on outcomes of Ewing sarcoma of the spine. Am J Cancer Res. 2018;8(7):1262‐1272. 30094099 PMC6079146

[3] Iacoangeli M , Dobran M , Di Rienzo A , et al. Nonmetastatic Ewing's sarcoma of the lumbar spine in an adult patient. Case Rep Oncol Med. 2012;2012:165289. 23133768 10.1155/2012/165289 PMC3485762

[4] Nair M , Sukumaran Nair R , Raghavan R , Parukkutty K , Sukumaran R . Primary Ewing's sarcoma of the spine in pediatric patients: a case series analysis and literature review. Middle East J Canc. 2015;6:115‐120.

[5] Boriani S , Biagini R , De Iure F , et al. Primary bone tumors of the spine: a survey of the evaluation and treatment at the Istituto Ortopedico Rizzoli. Orthopedics. 1995;18:993‐1000. 8584469 10.3928/0147-7447-19951001-09

[6] Boriani S , Weinstein JN , Biagini R . Primary bone tumors of the spine. Terminology and surgical staging. Spine. 1997;Phila Pa 1976(22):1036‐1044. 10.1097/00007632-199705010-00020 9152458

[7] Ilaslan H , Sundaram M , Unni KK , Dekutoski MB . Primary Ewing's sarcoma of the vertebral column. Skeletal Radiol. 2004;33:506‐513. 15232658 10.1007/s00256-004-0810-x

[8] Weinstein JB , Siegel MJ , Griffith RC . Spinal Ewing sarcoma: misleading appearances. Skeletal Radiol. 1984;11:262‐265. 6729498 10.1007/BF00351350

[9] Widhe B , Widhe T . Initial symptoms and clinical features in osteosarcoma and Ewing sarcoma. J Bone Joint Surg Am. 2000;82:667‐674. 10819277 10.2106/00004623-200005000-00007

[10] Turc‐Carel C , Philip I , Berger MP , Philip T , Lenoir GM . Chromosome study of Ewing's sarcoma (ES) cell lines. Consistency of a reciprocal translocation t(11;22)(q24;q12). Cancer Genet Cytogenet. 1984;12:1‐19. 6713356 10.1016/0165-4608(84)90002-5

[11] Sharafuddin MJ , Haddad FS , Hitchon PW , Haddad SF , El‐Khoury GY . Treatment options in primary Ewing's sarcoma of the spine: report of seven cases and review of the literature. Neurosurgery. 1992;30:610‐619. 1374853

[12] Indelicato DJ , Keole SR , Shahlaee AH , et al. Spinal and paraspinal Ewing tumors. Int J Radiat Oncol Biol Phys. 2010;76:1463‐1471. 19632062 10.1016/j.ijrobp.2009.03.042

[13] Boriani S , Amendola L , Corghi A , et al. Ewing's sarcoma of the mobile spine. Eur Rev Med Pharmacol Sci. 2011;15:831‐839. 21780553

[14] Tomita K , Kawahara N , Baba H , Tsuchiya H , Fujita T , Toribatake Y . Total en bloc spondylectomy. A new surgical technique for primary malignant vertebral tumors. Spine Phila Pa. 1976;1997(22):324‐333. 10.1097/00007632-199702010-00018 9051895

[15] Samartzis D , Marco RA , Benjamin R , Vaporciyan A , Rhines LD . Multilevel en bloc spondylectomy and chest wall excision via a simultaneous anterior and posterior approach for Ewing sarcoma. Spine Phila Pa. 2005;1976(30):831‐837. 10.1097/01.brs.0000158226.49729.6c 15803089

[16] Missenard G , Bouthors C , Fadel E , Court C . Surgical strategies for primary malignant tumors of the thoracic and lumbar spine. Orthop Traumatol Surg Res. 2020;106(1S):S53‐S62. 31843511 10.1016/j.otsr.2019.05.028

